# Usefulness of Melatonin and Other Compounds as Antioxidants and Epidrugs in the Treatment of Head and Neck Cancer

**DOI:** 10.3390/antiox11010035

**Published:** 2021-12-24

**Authors:** Joaquín Guerra, Jesús Devesa

**Affiliations:** 1 Otolaryngology, Medical Center Foltra, 15886 Teo, Spain; 2 Scientific Direction, Medical Center Foltra, 15886 Teo, Spain

**Keywords:** melatonin, curcumin, folic acid, epigallocatechin gallate, sulforaphane, sodium butyrate, epidrugs, epigenetics, cancer, antioxidants

## Abstract

Along with genetic mutations, aberrant epigenetic alterations are the initiators of head and neck cancer carcinogenesis. Currently, several drugs are being developed to correct these epigenetic alterations, known as epidrugs. Some compounds with an antioxidant effect have been shown to be effective in preventing these malignant lesions and in minimizing the complications derived from cytotoxic treatment. Furthermore, in vitro and in vivo studies show a promising role in the treatment of head and neck squamous cell carcinoma (HNSCC). This is the case of supplements with DNA methylation inhibitory function (DNMTi), such as epigallocatechin gallate, sulforaphane, and folic acid; histone deacetylase inhibitors (HDACi), such as sodium butyrate and melatonin or histone acetyltransferase inhibitors (HATi), such as curcumin. The objective of this review is to describe the role of some antioxidants and their epigenetic mechanism of action, with special emphasis on melatonin and butyric acid given their organic production, in the prevention and treatment of HNSCC.

## 1. Introduction

Head and neck squamous cell carcinomas (HNSCC), which include the oral cavity, oropharynx, hypopharynx, and larynx tumors, are a prevalent group of solid tumors, according to Global Cancer Observatory, there are 834,860 new cases and 431,131 deaths each year worldwide [1]. Unfortunately, the great majority of them are diagnosed in the locally advanced phase [2]. These patients generally are treated with multimodal treatments that include surgery, radiotherapy, and chemotherapy. Chemotherapy improved survival in non-metastatic HNSCC treated by surgery and/or radiotherapy (hazard ratio (HR) of 0.88) with an overall 4.5% benefit at 5 years. The benefit is more pronounced for concomitant chemotherapy as compared to induction chemotherapy, and there is no benefit for adjuvant chemotherapy [3]. In Europe, survival at 5 years is 54% for the youngest age group (15–45 years) and 35% for the oldest group of patients (over 75 years old) [2].

That is why the interest in the investigation of genes involved in the predisposition of HNSCC is not a recent phenomenon but a growing one. Along with the research of these genetic markers, there are antioxidants and epigenetic mechanisms of interest in the treatment of HNSCC; epigenetic mechanisms include, among others, DNA methylation, histone modifications, and changes in non-coding RNA [4]. MicroRNAs (miRNA) play an important role in tumorigenesis and may represent a novel panel of molecules for the development of cancer biomarkers with potential prognostic value to serve as a screening tool for HNSCC during the follow-up [5].

In recent years, interest has increased in developing drugs capable of reducing the deleterious effects of these genes, as antioxidants can do, or silencing them through epigenetic mechanisms. Despite the existence of multiple emerging studies demonstrating the in vivo and in vitro efficacy of these compounds, only a few have been approved for clinical use by the FDA, with limited results, especially in HNSCC [6].

In the case of epigenetic drugs, they can be classified into different groups: (i) DNA methylation inhibitors (DNMTi); (ii) histone deacetylase inhibitors (HDACi); (iii) histone acetyltransferases inhibitors (HATi); (iv) histone methyltransferase inhibitors (HMTi); (v) histone demethylase inhibitors (HDMi); (vi) inhibitors of protein binding to acetylated histone (PAHi); and (vii) inhibitors of protein binding to methylated histone (PMHi) [7]; nevertheless, many compounds do have combined effects, and potentially act on other epigenetic targets. Although monotherapy can activate these tumor suppressor genes or silence the activators, the main drawback of its individual use lies in its toxicity. Combination with other antineoplastic agents is a plausible option, with promising results when combining these inhibitors with chemotherapy or radiotherapy in HNSCC [8]. This is the case with drugs such as azacitidine, valproic acid, or 5-fluoro-2-deoxycytidine, but, according to clinical trials, the results are very limited at this moment [8]. Furthermore, some dietary supplements are epigenetically capable of modulating cancer [9], probably acting at least in part on the composition of the microbiota.

This review is justified by the appearance of new approvals in head and neck cancer (such as papilloma virus vaccine for HNSCC or the clinical trials with epigenetic drugs in HNSCC) [8,10]. Due to the appearance of this novel therapy and the absence of few reports in this regard, in this review, we will analyze the antineoplastic potential of some antioxidants, which can also act epigenetically, in the regulation of these tumors growth, putting a special focus on melatonin and butyric acid since they are produced by the body itself, and they play a very important role in the etiopathogenesis and treatment of many different types of cancer and many other diseases. Although their beneficial effects are explained leastwise by their antioxidant effects [11], their properties also include epigenetic modulation, antiproliferative effects (both preventive and therapeutic), or, as in the case of melatonin and other compounds, chemo- and radioprotective actions [12,13].

## 2. Carcinogenesis of Head and Neck Squamous Cell Carcinomas

The increasing knowledge of genetic and epigenetic mechanisms has promoted a better understanding of HNSCC, as multiple reviews have reported. For the development of HNSCC and premalignant lesions that induce their malignancy, several mutations have been described in genes that regulate cell proliferation, either by loss of their function or by their activation. We will now describe some examples of these mutations.

The inactivation of the p16/p14 locus (where genes associated with cancer control such as cyclin-dependent kinase inhibitor 2A (*CDKN2*A) and *ARF* are found) induces cell proliferation. Likewise, loss of function of the Notch receptor 1 (*NOTCH-1*) and *p53* genes prevents their tumor suppressor control. Particularly, *p53* is inactivated in a high percentage of subjects with HNSCC, reaching 80%, and is especially associated with alcohol and tobacco consumption, but not in cases of HPV infection. Moreover, its mutations are associated with a poor prognosis. On the other hand, activation of the Harvey rat sarcoma virus (*HRAS*) and phosphatidylinositol-4,5-bisphosphate 3-kinase, catalytic subunit alpha (*PIK3CA*) oncogenes involved in the RAF/MEK/ERK and PI3K signaling pathways have also been described. Other mechanisms of carcinogenesis involve the interruption of cell contact and differentiation pathways by loss of nuclear expression of RAR-beta (where there is inactivation of *Doc-1* gene), e-cadherin (*CDH1*), involved in cell adhesion and related to lymph node invasion, or metastasis-related degrading enzymes, known as matrix metalloproteinases (MMP). Finally, B-cell lymphoma-extra large (*BCL-XL*) and B-cell lymphoma 2 (*BCL-2*) are relevant anti-apoptotic genes involved in the interruption of the cell death pathway on HNSCC [14].

Regarding epigenetic mechanisms, it has been reported that histone deacetylases (HDAC) 1, 2, 8, and 9 are associated with advanced tumor stages, while methylation of H3K4 and H3K36 is related to active transcription and H3K27 me 2/3 and H3K9 me 2/3 to silenced genes [15]. Likewise, there are changes in DNA methylation in several genes associated with HNSCC. Examples of hypermethylated genes are *CDKN2A*, *O*-6-methylguanine-DNA methyltransferase (*MGMT*), Death-associated protein kinase 1 (*DAPK1*), Ras association domain family member 1 (*RASSF1*), cadherin-1 (*CDH1*), TIMP metallopeptidase inhibitor 3 (*TIMP3*), and phosphatase and tensin homolog (*PTEN*); examples of hypomethylated genes are peptidase inhibitor 3 (*PI3*) and programmed death-ligand 1 (*PD-L1*) [16,17]. Likewise, a report shows an increase in the expression of several miRNAs associated with chemoresistance [17].

## 3. Melatonin, a Pleiotropic Molecule

Before discussing how melatonin (MT) can be useful in the treatment of HNSCC, it is interesting to analyze how this molecule is produced and some of its main effects in organisms.

Among the physiological molecules that play many important roles in living beings, MT is very relevant because it appeared billions of years ago in evolution, and its primary structure has been conserved in all organisms (including plants and unicellular), but also, as the evolutionary process progressed, this molecule was developing many new and important properties. MT has been found in primitive bacteria (cyanobacteria and α-proteobacteria) [18], suggesting that it appeared to protect these early microorganisms from the high levels of O_2_ existing in the ancient atmosphere. The O_2_ released by the metabolism of photosynthetic bacteria appeared about a billion years earlier [19,20,21] and is responsible for the generation of extremely toxic reactive oxygen species (ROS) for cells. Therefore, it has been postulated that the appearance of MT was due to the need for a molecule capable of controlling oxidative stress by acting as an antioxidant and scavenger of free radicals in primitive photosynthetic prokaryotic bacteria [18,21,22,23,24,25]. As evolution continued, primitive bacteria were engulfed by the first prokaryotes, differentially evolving into chloroplasts (in cyanobacteria) and mitochondria (in α-proteobacteria). Consequently, all organisms were able to produce MT in these cellular organelles [22,23,24]. From these studies, it was concluded that MT appeared early in evolution to prevent or counteract highly toxic oxidative stress, which is why it has been conserved in all animal and plant species to date.

**Figure 1:**
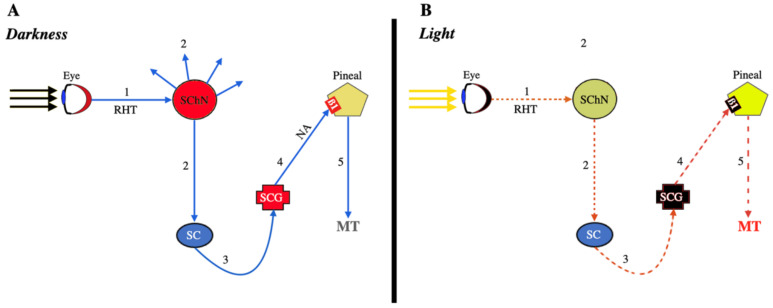
Signals involved in the production of MT by the pineal gland. (**A**): In the dark, the absence of light stimulation of the retina (red color) translates into signals (1) that stimulate the suprachiasmatic nucleus (SChN, red color) through the retino-hypothalamic tract (RHT). This nucleus then begins to send stimulating signals to different parts of the brain (2, blue arrows), but also a descending stimulating signal (2) to the spinal cord (SC, blue sphere), and from here to the adjacent sympathetic chain (3). These sympathetic signals reach the superior cervical ganglion (SCG), which is stimulated (red color) and sends stimulating signals (4) to the pineal, mediated by norepinephrine (NA) that interacts with a ß1-receptor in that gland (and also an α-adrenergic receptor, not shown), inducing the mitochondrial synthesis and release of MT (5). (**B**): In the presence of light, all the stimulating signals described above are interrupted (dotted red lines), and the superior cervical ganglion does not release NA; thus, the synthesis and secretion of MT are interrupted.

The effectiveness of MT as a free radical detoxifier relies on its ability to donate an electron (because of its high redox potential, around 0.73 V) or a hydrogen atom due to its special structure [26,27]. When MT interacts with different ROS, it produces cyclic 3-hydroxymelatonin and other metabolites [27,28,29,30,31], capable of acting as radical scavengers with even a greater capacity to neutralize ROS than MT itself [32,33]. Therefore, we could think of MT as an ancestral antioxidant, conserved throughout evolution and present in all cells of any living being, with the sole exception of red blood cells, because when these are formed in the bone marrow, they expel mitochondria. However, throughout evolution, the number of physiological functions of MT has increased significantly in multicellular organisms, so we can now speak of a pleiotropic hormone. Among these different functions, perhaps the one best known is, in most vertebrates but also in bacteria, that MT is simply a synchronizer between the dark/light cycle and sleep/wake rhythm. This synchronizing action is logical since more ROS are produced when there is activity, as occurs during the photoperiod when the metabolism is much more intense. In complex organisms, such as higher vertebrates, although melatonin is produced in practically all cells, the need for total synchronization with the outside led to the development of a specific gland, the pineal gland, capable of responding to the absence of light signals coming from the eyes, and sending this information to all the cells of the body so that they could have information about the dark/light cycle and modify their activity according to this cycle. Therefore, there is a neural connection between the eyes and the pineal gland, although this connection is quite complex, as shown schematically in Figure 1.

Briefly, the signals produced in the retina by the absence of light are sent through the retino-hypothalamic tract to a primary oscillator, the suprachiasmatic nucleus, which after being activated sends a series of signals to the brain but also to the spinal cord and from this to the sympathetic chain until reaching the superior cervical ganglion, which, in turn, when there is darkness, sends stimulating sympathetic signals to the pineal gland to induce the synthesis and release of MT into the circulation (Figure 1A). Conversely, photic stimulation of the retina leads to the interruption of this pathway, thus producing the abolition of the synthesis and secretion of MT (Figure 1B).

This is the most important mechanism involved in the production of pineal MT, mediated by norepinephrine (NA) that acts on α- and ß1-adrenergic receptors located in the pineal cell membrane. Stimulation of these receptors leads to an increase in the intracellular production of cAMP, which is ultimately responsible for the synthesis and release of MT. Therefore, MT secretion reflects the length of the scotophase, thus exhibiting a circadian rhythm. Although many other signals, produced by various neurotransmitters and other hormones, can affect MT synthesis and release, including the needed activation of protein kinase C (PKC) and increased Ca^++^, the sympathetic system plays a key role in this process. In fact, sympathetic denervation leads to loss of pineal gland function [34], while NA administration rapidly increases MT synthesis in these situations. At this point, it is important to note that the cervical region of the spinal cord is essential for normal secretion of pineal MT in humans [35]; therefore, this secretion is lost in tetraplegic patients [36,37] due to loss of sympathetic stimuli from the spinal cord to the superior cervical ganglion and from this to the pineal gland, as shown in Figure 1A. A similar loss occurs in pathologies that present with total blindness due to the lack of activation of the retino-hypothalamic tract.

Of interest is the finding that around 99% of the MT produced in vertebrates is not of pineal origin and is never released into the circulation; therefore, non-pineal plasma melatonin levels are very low. This reflects the existence of mitochondrial MT synthesis in practically all cells of the body (particularly in Harder’s gland, retina, immune system, ovary, testes, bone marrow, intestinal epithelium, etc.), where it acts as a scavenger of free radicals and as an antioxidant, but it also facilitates the processes that occur in the mitochondrial respiratory chain for energy production [38] and influences the release of cytochrome c [39]; furthermore, in the mitochondria, MT also stimulates the activity of superoxide dismutase (SOD2), an enzyme with antioxidant activity, and this induction involves an increase in the level of sirtuin 3 (SIRT3) [40], a protein that contributes to inhibition of oxidative stress [41] and also inhibits or reduces the activation of the NLRP3 inflammasome [42], in addition to fulfilling many other important functions in the body. The antioxidant effects of MT are also due to its induction of the expression of the main endogenous antioxidant enzymes (in addition to SOD): glutathione peroxidase, glutathione reductase, catalase, and the negative regulation of the expression of pro-oxidant enzymes, such as inducible nitric oxide synthase (iNOS), which prevents excessive production of peroxynitrite [43].

**Figure 2:**
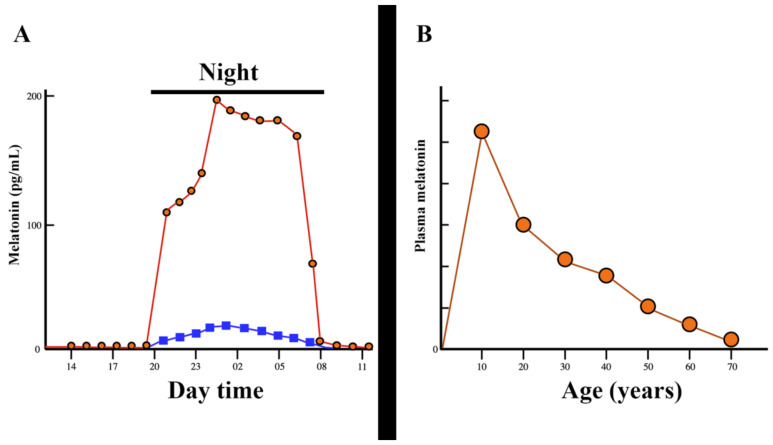
Evolution of melatonin secretion during the day and throughout life. (**A**): Plasma melatonin levels rise abruptly as the night begins, but this high increase comes from the pineal gland (red circles and red lines), while extra-pineal melatonin only experiences a small increase in plasma (blue squares and blue lines). When the darkness disappears, plasma levels of melatonin are virtually undetectable. However, even during high nocturnal secretion, a flash of 2000 lux sustained for one minute leads to the abolition of melatonin secretion (not shown in Figure). (**B**): The nocturnal secretion of melatonin is very low at birth, but then its pineal production increases continuously until it reaches its peak at puberty. From this age, the pineal production of melatonin undergoes a progressive decrease until it is practically undetectable in the elderly. (The red circles and red lines indicate the average amount of pineal melatonin production throughout life). Modified from reference [45].

Interestingly, despite the existence of a specific MT synthesis in mitochondria, these organelles are also a target site for the actions of the pineal or exogenously administered MT. However, the relationship between pineal or exogenously administered MT and the synthesis of this indoleamine in peripheral tissues seems to be tissue dependent [44]. Despite its very important physiological actions, the pineal production of MT is very low in the first years of age, after which a progressive increase in its production follows until the end of childhood. From that stage, already in puberty, the production of MT shows a continuous decrease as age increases until it is practically undetectable in the circulation of elderly people [45]. These changes are shown in Figure 2.

**Figure 3:**
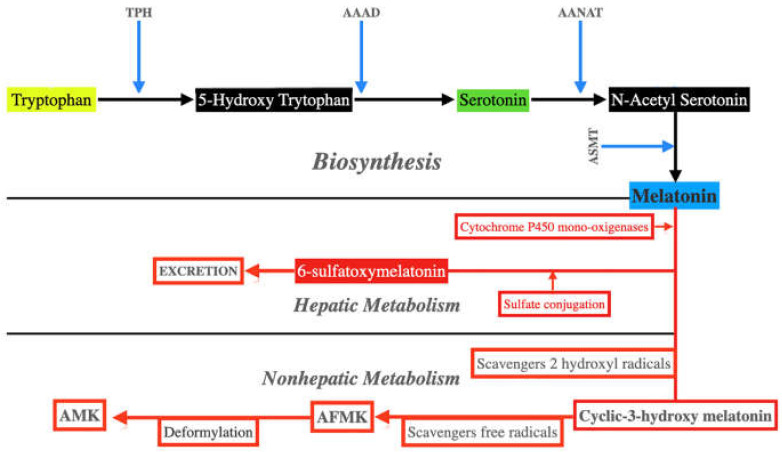
Synthesis and metabolism of melatonin. Melatonin biosynthesis first requires the hydroxylation and decarboxylation of tryptophan. This leads to the formation of serotonin, which, after being acetylated, is methylated and becomes melatonin. Melatonin metabolism takes place in the liver, from where it is excreted, and also in non-hepatic sites; in this case, the transformation of melatonin occurs non-enzymatically after free radical scavenging, giving rise to forms with even more powerful antioxidant activity than melatonin itself, such as cyclic 3-hydroxymelatonin and AFMK. In turn, AFMK can undergo deformylation resulting in the strong antioxidant AMK. TPH: tryptophan hydroxylase. AAAD: aromatic L-amino acid decarboxylase; AANAT: aryl alkylamine *N*-acetyl transferase. ASMT: acetyl serotonin *O*-methyltransferase. AFMK: *N1*-acetyl-*N2*-formil-5-methoxykinuramine. AMK: *N1*-acetyl-5-methoxykynuramine.

Basically, MT biosynthesis starts from tryptophan. This essential amino acid undergoes hydroxylation by tryptophan hydroxylase (TPH), which converts it to 5-hydroxy tryptophan. Then another enzyme, an aromatic L-amino acid decarboxylase (AAAD), decarboxylates 5-hydroxy tryptophan generating serotonin. Serotonin is then acetylated by aryl alkylamine *N*-acetyl transferase (AANAT) to form *N*-acetyl serotonin, which is finally transformed into MT by the action of acetyl serotonin *O*-methyltransferase (ASMT). Both AANAT and ASMT play a key role in the synthesis of MT. These reactions are shown schematically in Figure 3.

Plasma MT is metabolized primarily in the liver, where it is hydroxylated at C6 by cytochrome P450 monooxygenases and is then conjugated to sulfate and excreted as 6-sulfatexymelatonin (Figure 3). However, MT can also be metabolized in all cells by free radicals and many oxidants. In the first step, when it removes two hydroxyl radicals, MT is transformed into cyclic-3-hydroxy melatonin. MT oxidation in non-hepatic tissues leads to the formation of *N1*-acetyl-*N2*-formyl-5-methoxycinuramine (AFMK), the main metabolite of MT [46] (Figure 3). This is a very important mechanism because in this transformation of MT in AFMK, up to four free radicals can be eliminated, thus avoiding cellular oxidative stress. AFMK can be further metabolized to form *N1*-acetyl-5-methoxyquinuramine (AMK), which removes up to 10 free radicals and also downregulates and inhibits neuronal NO synthases [43].

The effects of MT appear after interacting with its receptors. In humans and also in animals, there are two main membrane receptors, MT1 and MT2, members of the G-protein-coupled receptor family, which induce different signaling pathways responsible for the effects of circulating MT in cells [47,48,49,50]. This is not the case for MT produced in the mitochondria. This intracellular MT is not released into the circulation, but it can act as a paracrine or autocrine factor after being released from the mitochondria and interacts with an MT1 receptor located on the outer mitochondrial membrane, most likely to induce or influence the release of cytochrome c [39]. Furthermore, MT can bind to specific sites in the cytosol [51] and in the cell nucleus [52,53,54]. In the case of the cytosol, MT binds to quinone reductase 2 (QR2), a detoxifying enzyme, also known as MT3 (receptor 3) [55], which reduces oxidative damage and calmodulin. The binding of MT to calmodulin has been related to the inhibitory effect of MT on cancer development [56,57]. In addition, nuclear receptor signaling is mediated by the transcription factor RZR/ROR, an orphan member of the nuclear receptor superfamily, postulated to be responsible for the anti-tumoral effects of MT [58,59].

Melatonin receptors mediate a number of intracellular effects, such as changes in intracellular cyclic nucleotides (cAMP, cGMP) and calcium levels, intracellular localization of steroid hormone receptors, and regulation of G protein signaling proteins. Interestingly, as with MT itself, its receptors and their responses show circadian variations. In fact, in some quite different pathologies, changes in the expression of these receptors have been observed [60].

In addition to acting as a synchronizer of the light/dark cycle with wakefulness/sleep and being a powerful antioxidant, MT has many other positive effects in humans: neuroprotective against some neurodegenerative diseases, anti-aging, heart protection, anti-inflammatory agent, protection of DNA and repair of damaged DNA, anti-angiogenic, oncostatic and oncolytic activity, decreases metastatic progression, exerts protective effects on the development of mucositis or dermatitis after chemo/radiotherapy, modulator of the immune response, protective effects in sepsis. Therefore, although the analysis of many of these actions of MT is not the objective of this study, once again, we have to consider MT as a very important pleiotropic hormone rather than as a simple synchronizer of the sleep/wake cycle between the organism and the environment, although this synchronization is key for the maintenance of normal human physiology. In fact, it is well known that the disruption of the circadian rhythm is associated with the appearance of diverse clinical disorders, including different types of cancer [61,62], particularly breast cancer in shift work women [62,63].

Interestingly, nasopharyngeal carcinoma has also been associated with disrupted circadian rhythm [64]. Precisely now, we will analyze the role of MT in a special type of cancer: squamous cell carcinoma of the head and neck.

### 3.1. Melatonin and Head and Neck Squamous Cell Carcinomas

Melatonin has been postulated to be a full-service anticancer agent [65]. Besides its antioxidant effects and the multiple mechanisms that MT induces for fighting against cancer and its metastasis, as we will see later, of high interest is the fact that MT exhibits a high ability to transform cancers resistant to chemo- or radiotherapy to a therapy-sensitive condition, perhaps by synchronizing or desynchronizing clock rhythms in cancer cells, therefore changing metabolic rhythms in these tumoral cells leading them to change their response to external stimuli and drugs [65]. Recently, two excellent reviews about the anticancer actions of MT have been published [44,66].

However, the possible therapeutic effects of MT in HNSCC have been little studied. The mechanisms by which MT acts as an antineoplastic agent in HNSCC are diverse, although mainly due to its antioxidant action. For example, fluctuations in endogenous MT secretion modulate malondialdehyde and superoxide dismutase levels after exposure to radiation therapy [67]. Indeed, high systemic and topical doses of MT are a protective agent against radio- and chemotherapy-induced damage to the oral mucosa [68,69,70]. Furthermore, its serum levels are inversely correlated with matrix metalloproteinase-9 (MMP-9), which is involved in the expansion and metastasis of this group of tumors [71,72]. An initial report showed that in patients with oral cavity cancer, MT levels were higher than in healthy subjects. The authors suggested that these findings could be justified by the insensitivity of its receptors [73]. However, a subsequent study reported that melatonin seems not to be involved in its pathogenesis, finding only a lower melatonin concentration in patients with lip, oral cavity, or pharyngeal cancer in the elderly, where melatonin synthesis and secretion are reduced [74].

The antioxidant function involves inactivation of the ROS-dependent Akt signaling pathway, downregulation of cyclin D1, PCNA, and Bcl-2, and upregulation of Bax, with the presence of hypoxia-inducible factor 1α (HIF1A) [75]. These effects condition the limitations of cell proliferation, including the epithelial-mesenchymal transition, as well as the conformation of microvascular channels induced by metastatic tumor cells [75]. Treatment with MT induces autophagy, as evidenced by increased expression of the biomarkers *LC-3B* and *Beclin-1* [76]. Increased autophagy appears to induce the MT2/mTORC1/TFE3 signaling pathway. Paradoxically, the inhibition of autophagy through the aforementioned pathway also seems to enhance the antitumor effect of melatonin, so the use of autophagy inhibitors could have synergistic effects in inhibiting tumor growth [77].

To avoid the effects of chemoresistance by rafampicin, it has been observed in animal models that the combination of this drug with melatonin leads to the suppression of the activation of the Akt/mTOR pathway, exerting a negative feedback loop from the specific descending effector from mTOR S6K1 activation to Akt signaling [78]. This adjuvant effect at high doses has been observed with other cytotoxic agents, such as cisplatin, and with radiation therapy [79]. In this and in a subsequent study using MT alone, an effect contrary to its antioxidant function has been observed, inhibiting glycolysis, resulting in increased ROS production, which leads to apoptosis and autophagia [79,80].

### 3.2. Epigenetic Effects of Melatonin on Head and Neck Squamous Cell Carcinoma

In oral cell carcinomas, epigenetic silencing of the melatonin receptor MT1 (4q35.2) gene may be a highly useful therapeutic mechanism [81]. MT decreases cell migration in OSCC, decreasing the expression of MMP-9 mRNA and protein metastatic factor. The reduction in *MMP-9* gene transcription is partially mediated by phosphorylation of the ERK1/2 signaling pathway, which regulates the expression of *MMP-9* transcriptional coactivators, such as CREB-binding protein (CREBBP) and E1A-binding protein p300 (EP300), and also decreases histone acetylation in HSC-3 and OECM-1 cells [82]. A similar effect of MT involving the *MMP-9* gene was observed in nasopharyngeal carcinoma, regulating the DNA binding activity of protein 1 (SP-1) of transcription factor specificity through the kinase c-Jun *N*-terminal/mitogen-activated protein kinase (JNK) pathway [83]. In addition, it exerts inhibition of lysine-specific histone demethylase 1A (LSD1) in xenografts and tumor cell lines derived from oral cancer patients [84]. In these cell lines, MT can reverse chemoresistance by positively regulating miRNA-892a and miRNA-34b-5p expressions, inducing apoptosis [85] and modulating other miRNAs, such as reducing miRNA-155 and increasing miRNA-21 expression, although its clinical significance is uncertain [86]. Another epigenetic antiproliferative pathway targets the miRNA-25-5p/NEDD9 pathway [87]. Along with the induction of apoptosis, MT can further induce Akt-mediated autophagy, p38, and JNK, as well as inhibit the expression of members of the ABCB1/ABCB4 ATP-binding cassette subfamily [85]. Melatonin also downregulates melatonin-regulated oral cancer stimulator (MROS-1), suppressing oral migration, refilling the protein homolog 2 (PRUNE). Cell migration induced by MROS-1, modulating PRUNE2, is mediated by an epigenetic mechanism regulated by DNMT3A [88]. Surprisingly, a recent article reported that high doses of MT could reverse its protective function toward a pro-oncogenic effect. High doses of MT may upregulate *FGF19* expression through activation of endoplasmic stress-associated protein kinase (ER), RNA-like endoplasmic reticulum kinase (PERK), eukaryotic initiation factor 2 alpha (eIF2α), the activating transcription factor 4 (ATF4) pathway, which in turn promotes FGFR4-Vimentin. This effect would negatively feed MT on its antitumor action and, in the long term, even induce metastasis [89]. These findings are contradictory given the ample evidence in the reports mentioned in this review, so further studies in this field would be desirable.

To date, only one clinical trial focusing on the effects of MT in HNSCC has been published. In this report, Kartini et al. showed that supplementation with 20 mg of MT associated with neoadjuvant treatment reduced the expression of miRNA-210 (mediated by ROS) and CD44, decreasing the percentage of tumor residues compared to patients treated with placebo and even neoadjuvant chemotherapy [90]. Despite these encouraging findings, the results were not statistically significant.

## 4. Other Antioxidants with Potential Epigenetic Effect on Squamous Cell Carcinoma of the Head and Neck

### 4.1. DNA Methylation Inhibitors (DNMTi)

#### 4.1.1. Epigallocatechin Gallate

Epigallocatechin gallate (EGCG) is one of the most important phenolic compounds. It is mainly found in green and black tea. Of all the catechins identified, EGCG is the one that exerts the greatest antioxidant effect through hydrogen peroxide and radical scavenger activity [91]. Along with its inhibition of cellular oxidation and prevention of free radical damage to cells, it inhibits DNMT1 and DNMT3A/3B activity in human cancer cell lines by binding and blocking DNMTs [7,92] and SIRTS [93]. The dose of this compound must be adjusted according to the genetic profile of the patient since high doses can cause hepatotoxicity [94].

Regarding HNSCC, EGCG has been shown to exert its antioxidant function in patients with this type of cancer, protecting the mucosa after radiotherapy in mouthwashes [95]. This antioxidant effect has also been seen in hair cell cultures in hair cells by inhibiting the Notch signaling pathway [96]. In fact, this and other tea derivatives have been studied in the prevention of oral premalignant lesions, observing that in patients who responded clinically to treatment, there were fewer cells of the epidermal growth factor receptor (EGFR) as well as downregulation of the expression of vascular endothelial growth factor (VEGF) and cyclin D1. However, the results did not differ significantly in terms of survival [97,98].

In relation to its epigenetic effects described in HNSCC, treatment with EGCG reverses, although only partially, the hypermethylation of the *RECK* gene (*reversion-inducing cysteine-rich protein with Kazal motifs* gene), significantly increasing the expression of its mRNA. It also decreases the levels of MMP-2 and MMP-9 metastasis-related proteins [99]. Furthermore, it has differential properties in normal and premalignant/malignant oral cells; in the latter, it acts as a pro-oxidant, inhibiting the expression of sirtuin-3 mRNA (SIRT3) through a specific decrease in the nuclear localization of the estrogen-related receptor α (ERRα), which is the transcription factor that regulates SIRT3 expression. Moreover, EGCG seems to differentially modulate the expressions of glutathione peroxidase and superoxide dismutase in normal and malignant oral cells. Therefore, it is easy to understand that the pro-oxidant effects of EGCG in oral cancer cells rapidly induce the appearance of mitochondrial reactive oxygen species that lead to cell death [93]. SIRT3 plays a dual role in cancer (as a tumor promoter or suppressor). This protein regulates proliferative and survival signaling pathways in normal and cancer cells by maintaining ROS thresholds, activating an antioxidant mechanism dependent on FOXO3 and NF-κB, and modulating them through MAPK/ERK, PI3K/Akt, and AMP kinase pathways [100]; some of these pathways have already been mentioned as associated with oncogenes in HNSCC [14]. Another report showed that EGCG increases miRNA-204 followed by a reduction in its Slug and Sox4 targets, thus inhibiting the epithelial-mesenchymal transition proliferation and metastasis in in vitro and in vivo tests [101]. It also inhibits the expression of β-catenin, whose signaling pathway is involved in different neoplastic processes [102]. Furthermore, the combination of EGCG with the epidrug erlotinib enhances the epidermal growth factor receptor (EGFR)-mediated signaling transduction pathways, previously mentioned, by increasing apoptosis, decreasing cell proliferation, and reducing pEGFR and pAKT [103]. Interestingly, another derivative of tea, gallic acid, exerts an inhibitory function against DNA methylation by reducing DNMT1 and DNMT3B in cell lines, acting, within a week, in the cell nucleus and cytoplasm [104].

#### 4.1.2. Sulforaphane

Sulforaphane (SFN) is the most characterized isothiocyanate. In addition to its anti-inflammatory and potent antioxidant properties, it protects DNA by modulating carcinogen metabolizing enzymes and blocks the action of mutagens; it also inhibits cell proliferation, angiogenesis, progression from benign to malignant tumors, and metastasis formation [105]. It can exert its antimutagenic effect even in foods that generate pro-oncogenic precursors, such as heterocyclic amines [106]. Epigenetic effects take place by inhibiting DNMT1, DNMT3A/3B, and tRNA methyltransferase [92].

SFN can act as a chemopreventive agent after exposure to nicotinic agents [107]. In addition, related to chemoprevention, it increases the protein expression of the transcription factor nuclear factor erythroid 2-related factor 2 (NRF2) [108]. This last activity would be mediated by upregulation of two genes, heme oxygenase 1 (*HMOX1*) and heat shock protein family A (Hsp70) member 1A (*HSPA1A*), as well as the MICA/B ligand of a natural killer cell activator protein, NKG2D [108]. In addition, SFN interferes with cell migration, downregulates cathepsin S expression through the ERK signaling pathway, and upregulates microtubule-associated protein 1A/1B-light chain 3 (LC3) [109].

In nasopharyngeal carcinoma cells, SFN inhibits growth through the DNMT1/Wnt inhibitory factor 1 (WIF1) axis [110], as well as inhibits total *STAT3* oncogene expression level and STAT3 phosphorylation (troy 704 and troy 705) by upregulation of miRNA-124-3p [111]. When combined with chemotherapeutic drugs, it interferes with proteins related to apoptosis through its antioxidant effects, downregulating *BCL2*, involved in the interruption of cell death [14], and upregulating *BAX* leading to an upregulation of the proapoptotic Caspase3, therefore enhancing its efficacy [112].

#### 4.1.3. Folate

Folates have been proposed as antioxidant agents with properties similar to those of other vitamins, such as C and E [113]. However, the effect of this vitamin B9 on cancer is controversial since while some reports associate it with protection, others describe that an excess exerts pro-oncogenic effects [114]. In the case of HNSCC, a large cohort study showed that folates play a protective role in its prevention [115].

There are very few reports on the epigenetic effects of folic acid in HNSCC, although this acid inhibits DNMT1 and DNMT3A/3B [92]. Interestingly, a decrease in dietary folate intake induces methylation of the cyclin-dependent kinase inhibitor 2A (*CDKN2A*) gene, which improves tumorigenesis and metastasis with cancer recurrence and poor prognosis; this relationship is modified by the methylenetetrahydrofolate reductase (MTHFR) genotype [116]. As can be seen, to date, only a protective effect has been demonstrated, so it remains to be demonstrated to what extent folate intake could exert an antitumor effect in patients with HNSCC.

### 4.2. Histone Deacetylase Inhibitors (HDACi)

#### Sodium Butyrate

Sodium butyrate (NB) is a salt of butyrate, a short-chain fatty acid with potential antineoplastic activity. Physiologically it is an important product of the microbial fermentation of dietary fiber in the large intestine. This small fatty acid has been shown to perform many important functions in the body. Its effects begin in the intestine, where it protects the epithelium, increases the production of mucin, and is the main source of energy to maintain its integrity and function. Therefore, its usefulness has been described in some intestinal pathologies, such as: ulcerative colitis, Crohn’s disease, irritable bowel syndrome, functional constipation, or diverticulosis [117]. In addition to these local effects, butyric acid is absorbed and has a number of other beneficial effects. Among them, it increases mitochondrial activity, exerts anticancer actions, improves insulin sensitivity, increases the function of the intestinal barrier, and shows potentially useful effects in various diseases (obesity, immunity, diabetes, and neurological disorders) [118]. Interestingly, disruption of the normal circadian rhythm has been shown to produce gut dysbiosis [119] that recovers after administration of exogenous MT [120], although MT synthesis also occurs in the gut. Therefore, the administration of MT may be useful in contributing to intestinal butyrate production, and both may fight against cancer.

It is likely that many of the beneficial effects of NB on different pathologies, including cancers, are due to its antioxidant properties regulating the cellular redox state by inducing the glutathione/glutathione S-transferase antioxidant system, which reduces ROS and allows the regulation of cell proliferation [121].

In relation to its epigenetic effects, NB binds competitively to the zinc sites of class I and II histone deacetylases (HDAC) [118]. This binding affects the hyperacetylation of histones, resulting in a modification of the DNA conformation, which consequently leads to the unwinding chromatin. The enhanced accessibility of chromatin to transcriptional regulatory complexes leads to increased transcriptional activation of several epigenetically suppressed genes. NB, as an HDAC inhibitor, induces cell cycle arrest in G1 or G2/M and also increases the expression of other genes and proteins involved in cell differentiation and apoptotic signaling [7]. Moreover, epigenetically, NB increases galectin-1 mRNA by, at least in part, the inhibition of histone deacetylation [122].

In vitro studies have indicated that NB has antitumor properties in HNSCC [123], with synergistic effects when combined with retinoic acid, increasing the expression of CDK6, p21, and p27, cell cycle regulatory proteins of the G1 phase, and inhibiting the expression of CDK2, the protein that regulates the cell cycle of the S-G2 phase in OSCC [124]. It also inhibits tumor angiogenesis by downregulating several growth factors, such as platelet-derived growth factor-B, angiopoietin-2, VEGF-C, and VEGF-D [125].

In thyroid carcinoma, in combination with another epidrug (decitabine), NB increases the messenger RNA levels of the thyroid sodium/iodide symporter (NIS), the global acetylation of histones, and increases the uptake of I^125^ by nine and eight times for DRO and two to seven cells, respectively [126].

### 4.3. Histone Acetyltransferases Inhibitors (HATi)

#### Curcumin

Curcumin is a yellow pigment isolated from the plant Curcuma longa. It exerts anti-inflammatory properties by inhibiting cyclooxygenases (COX) and other enzymes and blocks the formation of ROS; it also and disrupts cell signal transduction by various mechanisms, including inhibition of protein kinase C [127,128]. However, curcumin is an unstable compound with low bioavailability, which makes it necessary to explore approaches to counteract these weaknesses. These delivery approaches may include a micellar system, solid lipid particle, or hydrophilic nanoparticles [129].

Although curcumin has been described as an inhibitor of histone acetyl transferases, this pigment may also act as an inhibitor of histone acetylation and phosphorylation and as a DMNT blocker [7,92].

Based on the aforementioned effects, curcumin may play a role as an antineoplastic compound, inhibiting tumor cell proliferation and suppressing chemically induced carcinogenesis or tumor growth in HNSCC animal models [127,128]. Furthermore, and as with the rest of the previously reviewed compounds, a recent meta-analysis shows the beneficial effects of curcumin both in the prevention and in the treatment of radiotherapy-induced mucositis [130]. Moreover, a possible topical application for the prevention of oral carcinoma has been theorized on the basis that it would inhibit the inflammatory factors chemokine ligand 1 (CXCL1) and tumor necrosis factor alpha (TNF-α) [131].

Curcumin may act as an epigenetic drug in HNSCC by activating the ATM/CHK2 pathway and inhibiting nuclear factor-κB while increasing SIRT1 in a xenograft mouse model. The activation of this pathway and the inhibition of NF-κB (related to inflammatory processes and cancer) prevents tumorigenesis [132]. Curcumin also inhibits cell growth and progression by downregulating the PI3K-AKT-mTOR signaling pathway [133]. There is a synthetic derivative of curcumin, hydrazinocurcumin, a pyrazole obtained by cyclocondensation of the two carbonyl groups of curcumin with hydrazine, which has been reported to be the target of a nitric oxide signal-dependent inhibitory HAT, the histone acetyl transferase inhibitor VII (CTK7A), in oral cancer [134].

**Figure 4:**
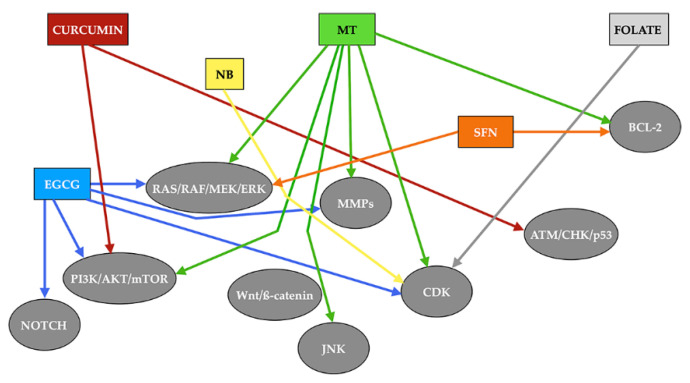
Signaling pathways stimulated by different epidrugs. Rectangles of different colors indicate each of the drugs analyzed, while arrows with the same color as the rectangle from which they indicate the signals induced by each of the drugs. MT: melatonin. NB: sodium butyrate. ECGC: epigallocatechin-3-gallate. SFN: sulforaphane. MMPs: matrix metalloproteinases. CDK: cyclin-dependent kinase. ATM: ataxia telangiectasia mutated. CHK: check point kinase.

Figure 4 shows a schematic description of the signaling pathways stimulated by the different compounds described in the text.

## 5. Conclusions

Table 1 summarizes the epigenetic effects of the antioxidants reviewed in this article.

Although the role of these antioxidants is limited, their adjuvant use with other cytotoxic agents makes them a very valuable tool as synergistic or useful agents to minimize the side effects of cancer treatment. Furthermore, these compounds exert oncoprotective effects. These effects are not only achieved by their own antioxidant role but also by other complex molecular mechanisms that regulate signaling cascades, inducing the expression of genes involved in tumor suppression. This induction of genes also includes epigenetic mechanisms of silencing or expression. Furthermore, this antineoplastic effect can be paradoxically exerted through a pro-oxidant action, inducing cell death. Among all the compounds analyzed, melatonin stands out for being a product manufactured in the body itself and its important pleiotropic actions. However, to date, despite the promising results of in vitro/in vivo studies, there is still little data to demonstrate the usefulness of the application of these compounds in HNSCC in humans.

## Figures and Tables

**Table 1:** Epigenetic effects of antioxidants in the treatment of HNSCC [7,40,64,84,85,86,87,88,92,93,99,101,104,110,111,116,118,122,132,134].

Drug		Effect	Target
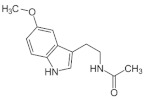	Melatonin	HDACi	SIRT1 SIRT3
		HDMi	LSD1
		miRNAs	miRNA-892a miRNA-34-5p miRNA-155 miRNA-21 miRNA-25-5p miRNA-210
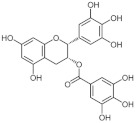	Epigallocatechin-3-gallate	DNMTi	DNMT1 DNMT3A/3B
		HDACi	SIRT3
		miRNAs	miRNA-204
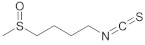	Sulforaphane	DNMTi	DNMT1 DNMT3A/3B Trn
		miRNAs	miRNA-124-3p
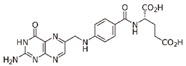	Folate	DNMTi	DNMT1 DNMT3A/3B
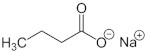	Sodium butyrate	HDACi	HDACI/II
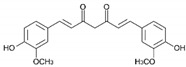	Curcumin	HATi	CTK7A CREBBP p300
		DNMTi	DNMT1
		HDACi	SIRT1
		Other (Histone phosporylation inhibitor)	GSK3β

HDACi: histone deacetylase inhibitor; DNTMi: DNA methylation inhibitor; HATi: histone acetyltransferases inhibitor (HATi); DNMT1: DNA methylation inhibitor 1; DNMT3A: DNA methylation inhibitor 3A; DNMT3B: DNA methylation inhibitor 3B; SIRT1: sirtuin-1; SIRT3: sirtuin-3; LSD1: lysine-specific histone demethylase 1A; Trn: tRNA methyltransferase; CTK7A: histone acetyl transferase inhibitor VII; CREBBP: CREB-binding protein; GSK3β: glycogen synthase kinase 3 beta.

## Data Availability

Data is contained within the article.
